# A Clinical Lecture on the Nature, Causes, and Treatment of Strabismus; Delivered at the Louisville Marine Hospital, February, 1842

**Published:** 1842-04

**Authors:** S. D. Gross

**Affiliations:** Professor of Surgery in the Louisville Medical Institute


### WESTERN JOURNAL

OF

### MEDICINE AND SURGERY.

---

APRIL, 1842.

---

### Art. I.—*A Clinical Lecture on the Nature, Causes, and Treatment of Strabismus; delivered at the Louisville Marine Hospital, February*, 1842. By S. D. Gross, M. D., Professor of Surgery in the Louisville Medical Institute.

Having recently performed the operation for strabismus several times in your presence, and many of you having expressed a desire to be made more fully acquainted with it, it is my design this afternoon to take up the subject in detail, and to lay before you such facts as an attentive study of this affection, during the last eighteen months, has enabled me to acquire.

Nothing, Gentlemen, so clearly evinces the happy progress of surgery as the remarkable improvements that have been effected, within the last six years, in the treatment of clubfoot and other analogous distortions. Until recently the nature and causes of these affections were, in great measure, if not wholly, misunderstood, and hence, as might have been expected, they were generally grossly mismanaged, the practitioner having no sound principles to guide or direct him. To Dr. Stromeyer, of Hanover, is justly due the merit of devising a mode of treatment at once easy and efficient, and which deserves allusion on this occasion, as it speedily led to the discovery of the operation which has lately been performed, with so much success, in various parts of the civilized world, for the relief of strabismus. When we reflect upon these curative procedures, their perfect simplicity, their entire freedom from danger, and their happy effects, it is only surprising that they should not have been revealed at an earlier period. Club-foot, wry-neck and strabismus must have been coeval almost with the human race, for it can hardly be supposed that mankind were ever wholly exempt from these and other infirmities incident to our nature. However this may be, their occurrence is exceedingly frequent at the present day, and the interest attached to them by the medical profession and society at large is fully commensurate with their importance. By the abnormal condition of a few muscles, often of very small size, or, indeed, of a single muscle, the most disagreeable distortion is produced; which, thanks to the researches of Stromeyer and others, may now be rectified in a very short time, or, as in the case of the eye, almost instantaneously.

The operation for the cure of strabismus was first performed on the human subject by Professor Dieffenbach of Berlin, in October, 1839. Prior to that period, however, its practicability had been tested by Mr. Anthony White of London, in several experiments on the inferior animals; and Dr. Stromeyer had also declared his conviction, as to its feasibility, from the fortunate results attending his cases of club-foot. The accounts of this novel operation were everywhere received with enthusiasm by the profession, and soon led to so many repetitions of it, that it may now be ranked as among the es tablished resources of surgery. By whom it was first executed on this side of the Atlantic, I do not recollect, nor is it necessary that I should; in the West it was first performed, I believe, by Dr. Goulding of Arkansas, and a few weeks subsequently by myself, Altogether, I have now operated between thirty and forty times.

Before I proceed to speak of the nature and causes of strabismus, and the operation calculated to relieve it, I deem it my duty to call your attention briefly to the consideration of the muscles and fasciæ of the eye, as a thorough knowledge of these structures is a matter of no little moment in reference to the subject before us.

Those of you who are familiar with the anatomy of the eye, are aware that it is under the control of six distinct muscles, by which its various movements are performed and regulated. Of these, four are straight, and two oblique, their names being derived from their position in the orbit. The former take their origin immediately around the margin of the optic foramen of the sphenoid bone, whence they proceed forward to the anterior portion of the scelrotic coat, one of them being situated at the upper part of the ball of the eye, another at the lower part, a third internally, and the fourth externally. In this course they lie in close contact with the surface of the organ, being connected to it by cellular substance; and, as they approach the anterior portion of it, they all terminate in small, flat, delicate tendons, which are inserted into the membrane just mentioned, at unequal distances from each other, as well as from the cornea. That this is true, my own researches, confirmed by those of my friend Dr. Buchanan, of Tennessee, and of Mr. Baker, an intelligent and enterprising member of the class, abundantly testify. This statement is at variance, I am aware, with that of the most distinguished anatomists both of this country and of Europe, a circumstance which only proves how imperfectly they have observed and studied this portion of the human system. Professor Horner, for instance, the most able writer on descriptive anatomy in the United States, fixes the distance at which the straight muscles are attached from the cornea at two lines for the superior and inferior, and at two or three for the internal and external; Mr. Quain, of London, indiscriminately at two lines and a half; Boyer, at two lines; while the more recent French authors, as Cloquet, Cruveilhier and Blandin, have evidently no settled notions in regard to the matter.

From the researches above alluded to, it was ascertained, beyond all doubt, that the internal straight muscle is not only more voluminous than the others, but that it is inserted somewhat nearer the cornea, the central portion of the tendon being rarely more than two lines distant from it, the superior margin more than two and a half, and the inferior more than three. This must give the muscle necessarily a great mechanical advantage, and is one reason probably, if not a principal one, why convergent strabismus is so much more frequent than the other varieties of the obliquity. The distance of the external straight muscle from the cornea may be stated at two lines and a half for the centre, three lines for the superior margin, and three and a half for the inferior; of the superior rectus, at three lines for the centre, three for the inner border, and four for the outer; and of the inferior, three for the centre, three and a half for the inner margin, and four and a half for the outer. To these admeasurements there are of course exceptions, and they are to be received, therefore, only in a general manner; for I have ascertained, to my perfect satisfaction, that the relative distances at which the straight muscles are inserted from the cornea, are liable to vary, not only in different individuals, but even in the eyes of the same person.

It has been already stated that the straight muscles are inserted at unequal distances from each other. This is true, so far as my own observations enable me to judge, in all cases, though there may be occasional exceptions. To exhibit this point in its true light, I will read you the following table drawn up from actual admeasurement:

| | | | | | | | |
|---|---|---|---|---|---|---|---|
| Distance between the superior and internal muscles | | | | | - | 3 | lines. |
| " | " | " | " | and external | " - - | 4 | " |
| " | " | " | external and inferior | | " - - | 3½ | " |
| " | " | " | inferior and internal | | " - - | 3 | " |

Such details may appear to you trivial and insignificant, and so, indeed, they are when considered merely in the abstract; but when viewed in reference to morbid action, or the effects of our surgical proceedings, they acquire a degree of interest and importance which those alone, who are familiar with the subject, can fully appreciate.

Are the straight muscles all of the same length, breadth and thickness, in other words, do they all possess the same degree of strength and development? It need hardly be said that they do not, though formerly the reverse of this was supposed to be true. The outer fasciculus is undoubtedly the longest; the inner the shortest and stoutest; the upper and lower being the smallest and nearly of equal length. Occasionally the disparity in the volume of these muscles is much greater than would, at first sight, appear possible. In one instance in which I made accurate admeasurements, I found the width of the internal bundle, one-third of an inch from its point of insertion, four lines, of the external three lines and a half, of the superior three lines and one-fourth, and of the inferior two lines and three-quarters. Generally speaking however, the difference is not so great, and it sometimes happens that the upper and outer muscles are the smallest.

Of the attachments of the oblique muscles it is not my wish to speak, as they are sufficiently well understood, and I have no new views to offer respecting them. I proceed, therefore, to make some remarks on what are denominated the fasciæ of the eye.

Until lately anatomists seem to have entirely overlooked these structures, since no notice of them is to be found in any of our treatises. Eight years ago, while engaged in teaching practical anatomy in the Medical College of Ohio, I satisfactorily demonstrated what I have since called the *ocular fascia*, and in my Elements of Pathological Anatomy, published in the autumn of 1839, is to be seen a succinct yet clear account of it, the first, I believe, ever presented to the profession. Six or eight months thereafter it was described by Dr. W. A. McDowell of this city, and it has likewise been fully noticed by Mr. Lucas, in his recent treatise on Strabismus, under the name of the *subconjunctival fascia.* To enable you to form a correct opinion of this matter, I beg leave to read the following extract from the work alluded to: "The connection between the conjunctiva and the sclerotica is established through the medium of a pretty thick layer of cellular tissue, which, from the character it plays in the diseases of the eye, deserves to be dignified with the appellation of the *ocular fascia.* When carefully dissected out, it is found to be semi-transparent, strong and elastic, disappearing gradually upon the posterior part of the ball. It is remarkably well developed in the horse and ox, and I have always succeeded in making it out distinctly in the human subject. Considered in reference to its functions, it is of the same use to the conjunctiva that the cellulo-fibrous tunic, so well described by Cruveilhier, is to the stomach and bowels. It is the exclusive seat, in most instances, of the vascularity which characterizes inflammation of the sclerotic portion of the conjunctiva and of the effusions attending it, whether of serosity, of lymph, of blood, or of pus."

To the preceding account I have nothing to add, excepting that the ocular fascia is a most important structure in regard to the successful issue of the operation for strabismus, that it is much stronger in old than in young subjects, and that it is intimately connected with the straight muscles, passing from one to the other, and thus forming partial sheaths for them.

The other fascia, equally important in a practical point of view, has been named by Mr. Lucas, who has furnished a very good description of it, the *submuscular*, from its situation. It lines the posterior surface of the straight muscles, and, as it extends towards their insertion, is reflected off from them upon the ball of the eye as far as the optic nerve. It thus forms a sort of pouch, or cul-de-sac, which encloses the adipous substance that exists in such abundance in this part of the orbit. In its structure, the submuscular fascia is somewhat stronger than the other, and consequently offers more resistance to the passage of a probe or other blunt instrument. Composed of cellular tissue, it passes from one muscle to the other, and is closely connected, in front, with the preceding, their substance being identified. The straight muscles are thus completely surrounded by these membranes, and hence, unless they are properly divided in the operation for strabismus, there must necessarily be a certain degree of failure, as the affected muscle will not be able to retract sufficiently to liberate the eye. Intending to revert to this point in a subsequent stage of the lecture, I pass on at once to the consideration of the more immediate object of our study.

Strabismus, or squint, as it is termed in common parlance, may be defined to be an aberration of the optic axes from their natural direction, by which the consent between the eyes is destroyed, and vision more or less impaired. The resultant deformity varies in different cases, from the slightest possible to the most disagreeable obliquity. The affected organ may be turned inwards or outwards, upwards or downwards, according to the muscle upon the derangement of which the squint depends. When the eye is directed inwards it constitutes what is called convergent strabismus; if, on the other hand, it inclines outwards, it is said to be divergent. The upward and downward obliquities have not received any particular names. As might be supposed, these different forms of strabismus do not occur with equal frequency. On the contrary, two of them are so exceedingly rare that I have not yet met with an instance, though I have examined the eyes of a very considerable number of persons affected with this infirmity. These two forms are the upward and downward, the latter of which is so seldom witnessed that it may well be doubted whether it ever occurs, except as the result of external violence.

The most common form of strabismus, by far, is the convergent, in which the eye is directed inwards, or inwards and upwards. Of five hundred and thirty-six cases collected from various sources by a writer in the Philadelphia Medical Examiner, five hundred and six were of this description; a proportion which fully corresponds with my own but more limited experience. The degree of obliquity may be very slight, or so great that when the person looks directly forward with the sound eye, the cornea of the other shall be almost concealed at the inner canthus. It is worthy of remark that in this variety of the lesion, at least so far as my observation extends, the organ never inclines downwards, but almost constantly somewhat in the opposite direction.

Next in point of frequency is the divergent variety, but even this, in comparison with that just described, must be regarded as quite rare. Of eight hundred and sixty-six cases reported in the work above referred to, it was witnessed only forty-four times, and thus far I have myself seen only three or four examples of it. The eye in this form of strabismus is rarely drawn out very far, nor is it so apt to be attended with the same amount of upward obliquity as the convergent.

All writers on strabismus appear to think that, in the great majority of cases, only one organ is affected. Thus in the article in the Philadelphia Examiner, it is stated that the distortion occurred four hundred and fifty-nine times in one eye, and only forty-seven times in both. Dr. Dix, of Boston, in a small treatise on strabismus, makes a similar remark. Of fifty cases, which fell under his observation, the lesion is said to have been limited to one eye in thirty-six. Now I am convinced from a good deal of experience that nothing can be more unfounded than this opinion; which is so much the more to be deprecated, because it is calculated to lead to very serious errors in practice. I unhesitatingly assert that in nearly all instances, at least of convergent squint, both eyes are implicated, though not in an equal degree. Usually one is more affected than the other, which the patient considers as his good eye, as it is the one which he constantly employs in viewing objects. Nor is it at all surprising that this should be the case, when we reflect upon the remarkable sympathy existing between these structures, and upon the fact that when one is diseased, the other is very liable to take on morbid action also. Amaurosis in one eye is very frequently followed by a similar malady in the other; the same is true of cataract and some other affections. In the natural state there is a perfect agreement between the optic axes, produced by the harmonious action of the straight muscles; but when this consent is destroyed, as it is in strabismus, the eyes lose their parrallelism, and their movements become partially involuntary, especially those of one eye. Be this, however, as it may, I feel certain that, if you will examine this matter for yourselves, you will be satisfied of the truth of what has been here said. The subject, it will be perceived, is one of paramount importance in respect to the results of our curative measures; for if, when both eyes are involved, we were to operate only on one, under an impression that the lesion was entirely limited to it, our success would necessarily be imperfect, or the attempt perhaps a complete failure.

I have already said that one eye is ordinarily more affected than the other, and this, if I mistake not, will be found to be the left in three cases out of five. Such, at all events, is the result of my own experience, which differs somewhat, perhaps, from that of others. Mr. Lucas thinks that the proportion in favor of the left eye is as three to two; and Dr. Phillips, of Leige, maintains that the right organ is much more frequently involved than the other. The statistics in regard to this question are, however, still very imperfect, and not a little additional observation is necessary before it can be finally settled.

It rarely happens that both eyes become deranged simultaneously; on the contrary, one generally squints first, and after a while the other, the interval being probably very short.

Whether strabismus occurs with equal frequency in both sexes, is a point for the determination of which we have no accurate data. Of thirty-three cases on which I have opera ted, only five were females, whereas in the fifty cases published by Dr. Dix of Boston, only nineteen were males; thus exhibiting a most marked disparity in relation to the subject under consideration. The difference is perhaps not great either way, but as it is of no moment, in a practical sense, I need not dwell upon it longer in this place.

I come, secondly, to make some remarks on the exciting causes of strabismus. These, as might be imagined, are extremely numerous and diversified. One of the most frequent is imitation. Nearly a seventh of all the cases that occur are probably thus induced. Hence our school-rooms are a fruitful source of mischief in this way, one cross-eyed child being often the cause of strabismus in many others, simply from that habit of imitation to which all young persons are so much addicted. Ophthalmia, however induced, is another and a very common cause of strabismus. I have seen a number of instances of this kind, and others are mentioned by different authors. Convulsions, eruptive diseases, such as measles and scarlet-fever, hooping-cough, derangement of the digestive organs, injury on the eye, and difficult dentition, may all be enumerated as so many causes of the affection in question. Oftentimes it arises without any assignable reason, and when the person is in the enjoyment of the most perfect health. To place this subject in a more tangible form, I beg leave to present the following table, compiled from the writings of Melchior, Lucas, Duffie, Ammon, Phillips, and others, the number of cases being three hundred and twenty-three: *

| | |
|---|---|
| Imitation, - - - - | 61 |
| Ophthalmia, - - - - | 36 |
| Convulsions, - - - - | 31 |
| Looking fixedly or suddenly at objects in youth, | 16 |
| Injuries inflicted on the eye, - - | 15 |
| Measles, - - - - | 14 |
| Hooping-cough, - - - | 11 |

* Philadelphia Medical Examiner, vol. iv, p. 439.

| | |
|---|---|
| Small pox, - - - - | 11 |
| Injury on the head, - - - | 10 |
| Looking at objects on the eye-brow, nose, inner canthus, and thumb while held in the mouth, - | 9 |
| Difficult dentition, - - - | 8 |
| Falls and blows, - - - | 7 |
| Inflammation, - - - - | 5 |
| Excessive fright, - - - | 4 |
| Exposure to light and heat of the fire in infancy, | 3 |
| Intestinal worms, - - - | 3 |
| Imperfect cataract, - - - | 3 |
| Holding the head sideways while knitting, - | 3 |
| Epilepsy, - - - - | 2 |
| Looking at the sun, - - - | 2 |
| Amaurosis, - - - - | 2 |
| Burns of the abdomen, - - - | 2 |
| Chorea, - - - - | 1 |
| Severe whipping, - - - | 1 |
| Watching the motion of a shuttle, - - | 1 |
| Fit of crying - - - - | 1 |
| Concussion of the brain, - - - | 1 |
| Fracture of skull, - - - | 1 |
| Ill health, - - - - | 1 |
| Supposed congenital, - - - | 17 |
| Unknown, - - - - | 41 |
| | 323 |

Of the above cases, seventeen, it will be perceived, are supposed to have been congenital, a circumstance admitting, I think, of some doubt. Without altogether denying the possibility of this occurrence, I am inclined to believe that many cases, considered as congenital, are in truth not at all of this description, but that they arise soon after birth, without any obvious cause, and without any knowledge on the part of the parents or friends of the infant as to the precise period of the commencement of the lesion. Judging from what we know of club-foot, strabismus may unquestionably be a connate affection, and such it certainly is in some instances; all I wish to impress upon your minds is, that it is much less frequent, in my opinion, than is generally supposed.

Some writers imagine that there is occasionally a hereditary predisposition to strabismus. This is doubtful. If we sometimes meet with cross-eyed children, whose fathers or mothers are laboring under the same affection, it by no means proves that the distortion was transmitted to them in the manner in which certain maladies are transmitted by the parent to his offspring. It only shows a coincidence, which may be explained, in the generality of cases, on the assumption that the children have acquired the distortion by imitation or by some other cause, not that it was entailed upon them prior to birth. In the same manner we may satisfactorily account for the presence of strabismus in several members of the same family, of which a remarkable instance has recently been brought within my own knowledge. Of three brothers, one has three children affected with strabismus, the other two, and the third one. The parents have perfectly sound eyes, and so have the uncles and aunts, except one, on whom I operated successfully on both organs several months ago. Last November I operated for cataract on three children, belonging to a gentleman from Mississippi, who informed me that of six others at home, three were affected with strabismus. Both he and his wife, as well as their immediate relations, are free from the affection.

Strabismus essentially consists in a morbid contraction of one or more of the muscles of the eye. This, as we have already seen, is usually the internal rectus. The shortening, which varies according to the extent of the squint, always leads to a corresponding elongation of the opposite muscle, so that it gradually loses, either wholly or in part, its antagonizing influence. But few post-mortem examinations have been made of the eyes of persons laboring under strabismus; nevertheless, they have been sufficient to show that there is generally a marked difference between the straight muscles, the one affected being not only wider and thicker than the rest, but also of a deeper color. These changes were well seen in the case of a female who died at the Hospital last summer, under the care of my friend Dr. Lewis Rogers. She was upwards of thirty years of age, and had been affected with convergent strabismus from an early period. The left eye was more distorted than the right, and the internal straight muscle of that side was, as I ascertained by a careful examination, proportionably larger than the other. This, it is true, might have been a mere coincidence; but, supposing that it was not, and that the difference under consideration really exists, as a general rule, the question occurs, is this undue development the cause or the effect of the strabismus? This is a matter which is not so easily disposed of as might at first be imagined. As to myself, I presume that most cases of this affection are occasioned originally by some perverted action of the nerves which supply the muscles of the eye, rather than of any actual lesion of these fleshy bundles themselves. What the precise nature of this morbid action is has not, and probably never will be, determined; but it is reasonable to infer that it will be followed, sooner or later, by perverted action of one of the muscles at least to which I have alluded, and which must thus, by degrees, obtain a powerful ascendency over all the rest. The resultant distortion becomes permanent, and the affected muscle, constantly occupied in holding the eye in its unnatural position, acquires a corresponding degree of development; in accordance with a law of the animal economy that, in proportion as an organ is exercised, so will be its size and strength.

One of the most unpleasant effects of strabismus is the disagreeable deformity which accompanies it, by which the individual is rendered an object of remark and ridicule. Were this confined to infancy and childhood, no evils would flow from it, but when we reflect that it continues during life, and that it is a source of constant annoyance and mortification, the influence which it exerts upon the temper of the sufferer must often be of the most unhappy kind. On this account alone, if on no other, we cannot be too grateful to Stromeyer for having devised, and to Dieffenbach for having first practised, an operation fraught with so much blessing to a class of beings whose cases were usually regarded, until recently, as beyond our resources. But there is another effect, if possible, of a still more serious character. I refer to the impairment of the vision of the affected eye. This, which is never wholly absent, always varies with the extent of the deformity, and the length of time that has elapsed since its occurrence. In some instancs, especially in those of long standing, the sight is altogether destroyed, the retina being as insensible as in confirmed amaurosis. In another series of cases, the person is myopic, or sees objects only at a short distance. In a third series the vision, perhaps, is double, or objects appear indistinct, or run into each other, the image painted on the retina being confused and imperfect.

It is a well-known fact that strabismus has no tendency towards a spontaneous cure. On the contrary, it generally manifests a disposition to increase, and this is particularly the case in children of a nervous, excitable temperament. Indeed, the very worst forms of squint I have ever seen occurred in persons of this description. The question, then, arises, at what age ought we to operate? My opinion decidedly is the sooner the better. Provided the child is in good health, and not under a year of age, I would not hesitate a moment to resort to the knife for its relief. And why should we? The operation itself is not particularly painful, and if it be attempted at an early period it will commonly be necessary to perform it only on one eye, whereas if it be deferred until the age of twelve or fifteen, as has been suggested by some, we cannot possibly get along without dividing the corresponding muscle of the opposite side. Moreover, the sight in the meantime will become considerably impaired, and the patient be an object of ridicule and insult; both of which may thus be avoided. But there is another, and, as I conceive, a very strong reason, for the early performance of the operation. We have already seen that the ocular and submuscular fasciæ are much more delicate and elastic in infancy and childhood than at the subsequent periods of life, and they will not therefore, be so liable, if they are not extensively divided, to interfere with the success of the operation. But it may be urged that a resort to the knife at this tender age will be both difficult and dangerous; difficult because of the struggles of the little patient, and dangerous because of the great susceptibility of the nervous system. In regard to the first of these points it may be stated that the resistance will never be so great but that it may be surmounted by proper management; and, as respects the other, it is well known that it has been vastly overrated. Much severer operations are frequently performed at a much earlier period. I have seen the common carotid artery tied, successfully, in an infant of six months, and have myself repeatedly operated, with similar results, for hare-lip, and that too in the very worst forms of that malformation, within the first year. I do not, therefore, in these objections, discover any adequate reason for postponing the division of the affected muscle.

The instruments which I employ in this operation are two lid-holders, a double, sharp-pointed hook for fixing the eye, a pair of forceps for pinching up the conjunctiva, and a scalpel or pair of scissors for dividing this membrane, the two fasciæ, and the affected muscle. The lid-holders (fig. 1) are each about six inches long, made of steel, quite slender, and slightly curved at the extremity, which is perfectly smooth and polished, constructed after the manner of a fenestrated speculum, and not more than four lines wide. These instruments may be conveniently replaced by a common speculum and the finger of an assistant; still, they are very useful, and I prefer them to any other contrivance I have seen. The hook for fixing the eye is similar to that contained in our eye-cases, (fig. 2). It should not exceed five inches in length, and should be provided with a moveable slide, so as to admit of the proper separation of the branches, each of which, two lines wide, terminates in a short hook as delicate as the finest needle. The forceps should also be rather small; and the blades of the scissors, curved on the ege, should form an angle of about 35° with the handle; they should be very narrow, and their extremities only moderately sharp. Until lately I was in the habit of employing a knife instead of the scissors, and also a curved director for elevating the muscle previously to dividing it; but as this procedure is painful, and does not expedite the operation, I have abandoned it. I now use the director (fig. 3) only to ascertain the extent of the incisions, and in this respect it is really a valuable instrument, though a common probe will answer just as well.

Previously to commencing the operation, the patient should be placed on a table of convenient height, covered with a blanket, and the head and shoulders elevated with pillows. His face, lying horizontally, should look towards the light, which, however, for obvious reasons, should not be too strong; and the sound eye be covered with a bandage or handkerchief, to enable him the better to roll the other outwards. If the surgeon is ambidexter it does not matter where he stand; but if he uses one hand more adroitly than the other, he should place himself on the right side when he wishes to operate on the left eye, and, conversely, on the left, if he desires to operate on the right. Only two assistants are necessary; one of whom, standing at the head of the patient, elevates the upper lid, and holds the eye by fixing the sharp hook into the sclerotic coat, about two lines behind the cornea; the branches should be separated one third of an inch, and the interval between them should accurately correspond with the horizontal diameter of the eye. This precaution should never be neglected, otherwise it will not be so easy to find the affected muscle. The points of the hook should be fairly implanted into the substance of the sclerotic tunic, but no more. If it be passed simply through the conjunctiva, it will be impossible to steady the eye, to say nothing of the danger of lacerating that membrane, and so inflicting unnecessary pain upon the patient. On the other hand, if it be pushed through the sclerotic coat, serious mischief, eventuating in destructive inflammation, might be the consequence. The other assistant, placed on the side of the affected eye, depresses the lower lid, and hands the sponges to the operator. It is sometimes more convenient to let this assistant steady the eye.

Every thing being thus prepared, the operator pinches up a small fold of the conjunctiva immediately behind the hook, or, in other words, about two lines from the cornea, and makes a slight vertical incision through it with the knife or scissors, as he may prefer. Relinquishing his hold with the forceps, the edges of the wound will be found to retract, so as to be at least from four to six lines in length by two or three in breadth. At this moment there is usually some degree of hemorrhage, amounting occasionally to more than half a tea spoonful, especially if the incision has been made too far back, near the semi-lunar valve, where the parts are always more vascular than further forward. To arrest this, a small sponge, pressed out of cold water, should be repeatedly applied; or, if it prove troublesome, the operation may be entirely suspended until it ceases. The ocular fascia is now divided, and, as soon as the muscle (fig. 4) is fairly exposed, it is cut across with the scissors, one of the blades of which is passed behind it. The moment this is accomplished, the eye, from the traction exerted upon it by the hook, springs towards the opposite side, and the muscle retracts within its sheath, especially if it has been thoroughly liberated from it connexions with the ocular and submuscular fasciæ. To effect this, which I regard as of paramount importance, the scissors should be passed for some distance around the eye, nearly as far, indeed, as the margins of the adjacent straight muscles.

As soon as the muscle is fairly divided, the eye usually resumes at once its natural position in the orbit, moving, if the other be sound, in perfect harmony with it. Occasionally, however, it retains some degree of its original obliquity; in which case it becomes necessary to re-apply the instruments with a view of ascertaining the cause of the malposition. This will generally be found to depend upon an imperfect division of the muscle, or of one or both fasciæ, by which the muscle is prevented from retracting sufficiently within its sheath. In some instances it remains without any assignable cause, but rarely beyond a few minutes, or, at farthest, a few hours.

The operation being completed, the eye is bathed in cold water, to free it of blood, and the patient is confined for a few days in a dark chamber. Light diet should be enjoined, and, if inflammation arise, recourse must be had to antiphlogistic measures. Bleeding can seldom be demanded; thus far I have not been obliged to resort to it in a single instance, a dose of salts, oil, or calcined magnesia being all that was required, and in some even this was not necessary. As a local application, cold or tepid water, as may be most grateful to the part, is amply sufficient. Occasionally there is a good deal of pain soon after the operation, with more or less constitutional disturbance, such as slight shivering, headache, and nausea; these symptoms are best relieved by warm drinks and an opiate. The ecchymosis which attends this operation, and which is sometimes considerable, requires no particular treatment: no inconvenience arises from it, and it commonly disappears in a few weeks. I have never known suppuration or abscess to follow this operation, but several cases are related by writers. Such an occurrence implies unusual violence, which cannot be too much condemned. The same remark is applicable, still more forcibly, to the division of the sclerotic coat, and the partial escape of the humors of the eye; an accident which has happened in the hands of some ignorant bunglers. For five or six weeks after the operation the patient should refrain from reading and looking at minute objects. By neglecting this precaution chronic inflammation, with weakness of sight, may be induced and kept up for several months, much to the annoyance of the sufferer.

It may not be uninteresting briefly to inquire into the process employed by nature to repair the wound made in the operation. From the distance at which the edges are separated from each other, it is obvious that it cannot heal by union by the first intention; nor have we any means of remedying this difficulty, since it is impossible in parts so delicate as those under consideration to employ the suture. Soon after the operation is over, the margins of the incision become coated with coagulating lymph, which is sometimes effused in such quantities as to give rise to considerable pain and a sensation like that produced by the presence of some foreign body. The vessels in the surrounding structures are somewhat enlarged, there is more or less lacrymation, and the lids feel stiff and uncomfortable. The sclerotic coat at the bottom of the wound remains visible for five or six days, when it becomes covered with granulations, which, uniting with those of the sides, gradually fill up the breach, the whole process, from the commencement to the completion of the cicatrization of the conjunctiva, occupying from two to four weeks.

Occasionally, though not always, the process of cicatrization is retarded by the presence of fungus granulations. When this is found to be the case, they should be snipped off with a pair of scisors, a proceeding far preferable to the application of the nitrate of silver, which is not only more painful, but less effectual.

It has been recommended by some surgeons that as soon as the soreness occasioned by the operation has subsided, the patient should begin to turn the eye outwards, and that these efforts should be daily persevered in, until it regains its natural position in the orbit. In my early cases, before I had devoted much attention to the subject, I adopted this advice, but the result in every instance disappointed me. Nor do I now perceive any good reason for following it, persuaded as I am that it is not founded upon correct principles. Where the eye still retains some degree of obliquity after the operation, it may be positively assumed that the section of the affected muscle, or of its investing fasciæ is imperfect; how then, when this is the case, can we look for success? Again, the eye operated on may be entirely straight, and yet not move in concert with the other. This I have witnessed repeatedly, and hence my invariable rule, under such circumstances, is, to divide at once the corresponding muscle of the opposite side, for the reason already mentioned, in a former part of this discourue, that the distortion almost invariably involves both organs.

The operation for strabismus is liable to occasional failure, the principal causes of which may be thus enumerated: 1. Imperfect section of the affected muscle or of the ocular and submuscular fasciæ. To this subject I have already several times adverted, and I need therefore only further remark concerning it that the operator should never neglect to divide these structures most thoroughly. In bad cases the scissors must be carried up and down as far as the contiguous straight muscles, so as to denude completely the sclerotic coat for more than a third of its circumference. The fasciæ must be effectually raked up, otherwise it will be impossible for the muscle to retreat within the orbit. 2. Excision of a portion of the conjunctiva, eventuating in contraction of this membrane during the process of cicatrization, may be considered as another cause of failure. As there is no necessity whatever for such a proceeding, since it does not in the slightest degree facilitate the operation, I need scarcely say that it should be most studiously avoided. 3. Strabismus is sometimes complicated with other diseases, such as convulsions, epilepsy, hydrocephalus, and similar lesions. When this is the case the operation cannot be performed with any prospect of success, and had better be declined altogether. The existence of amaurosis does not necessarily lead to failure; if cataract be present, it should be broken up either at the time of the operation or before. 4. But the most powerful cause of all in my opinion, and one which has not been sufficiently insisted upon by writers, is the co-existence of strabismus in both eyes, and the fact that our operative procedures are usually limited to one of these organs. Now this is at direct variance with good practice and common sense. In several instances in which only partial success attended my efforts, the cause was entirely ascribable to this circumstance, and so thoroughly am I persuaded of its importance that I have laid it down, as a rule, never to operate on one eye only when it is certain both are affected. The only exception to this is where the patient is very young, when the section of a single muscle will sometimes suffice, though even then not always. 5. A fifth cause of failure is the re-adherance of the posterior extremity of the muscle to an unfavorable point of the sclerotica, by which it is again enabled to exert an undue influence over the movements of the eye. The manner of obviating this difficulty has been already indicated, and I need not therefore dilate upon it in this place. These, I believe are the principal circumstances which interfere with the success of the operation; there may be others, but they are unimportant and such as will readily suggest themselves to your minds.

I come now to speak, in the last place, of some of the effects of the operation. These may be considered, first, in relation to the deformity or obliquity of the organ; secondly, in respect to the sight; and lastly, in relation to the prominence of the eye and the separation of the lids.

When the operation has been well executed, the deformity, as was before mentioned, disappears either instantaneously, or, at all events, in a few hours, and the organ, there is reason to believe, ever afterwards remains straight. It is true causes may subsequently arise which may lead to a relapse, or return of the obliquity, but this must certainly be very rare, and is to be regarded altogether as an exception to the general rule.

The effect upon vison is at first rather disagreeable than otherwise; at least this is true in some instances. It is only by degrees that the affected organ regains its function, and in many cases a considerable period must necessarily elapse before this is fully brought about. Occasionally, in fact, the retina, from long disuse, or other causes, is so completely paralysed that the sight is never restored, and it is in cases of this description that we should always look for a slight return of the distortion. Another effect, and that a very unpleasant one, which is sometimes witnessed, is double vision. This is evidently dependent upon a want of agreement between the optic axes, and seldom lasts beyond a few days, unless the distortion has been but imperfectly remedied.

The only other effect which I shall notice as attendant upon this operation is a peculiar prominence of the eye. It is generally very well marked, though not equally so in all cases, and imparts to the organ a full, bold expression; it is accompanied by a considerable separation of the lids, and is caused by the liberation of the eye from its confined state.

In speaking of the operation for strabismus, I did not deem it necessary to give you an account of the various plans that have been suggested by other surgeons; I pointed out to you the mode which I have myself been in the habit of pursuing, and which I can confidently recommend to your notice, not only as safe and expeditious, but as effectual perhaps as is compatible with the nature of the affection which it is intended to remedy. There is one method, however, to which I must briefly refer, as well on account of the high authority from which it emanates, as on account of its supposed advantages. I allude to to the *subconjunctival plan*, as it is termed, first devised by Mons. Guerin, the celebrated orthopedic surgeon of Paris. The manner of performing this operation in convergent strabismus is as follows: The patient lying on a bed or sofa with his head somewhat elevated, an assistant separates the lids, while the operater inserts the double hook in the same manner and at the same distance from the cornea, as in the ordinary method. Another assistant with a delicate hook lifts up a fold of the conjunctiva, midway between the cornea and the semi-lunar valve, when the surgeon, armed with a lancet-shaped scalpel, makes an aperture into that membrane on a line with the inferior margin of the muscle, carrying the point at the same time backwards to open the investing fasciæ. Another knife,* (fig. 5), peculiarly adapted to the operation and which you here see, is then carefully insinuated beneath the tendon with the side of the blade resting nearly flat upon the sclerotic coat, when, turning the edge forward, the division of the structure in question is easily effected, being attended, it is said, with a distinct snapping sound.

---

*For this instrument I am indebted to my friend and former pupil, Dr. Charles A. Pope, who spent several years in Paris, and who is now settled in St. Louis.

Having never operated according to this method, I can not of course say anything about it from my own observation. Dr. Davenport, of Boston, who has executed it in two cases, thinks it posesses strong claims to a more extended trial, and enumerates the following supposed advantages: First, there is little or none of that unpleasant gaping at the inner canthus which sometimes so much disfigures those who have undergone the common operation; and secondly, there is less risk of severe inflammation, or suppuration, the patient, from the absence of an open wound, being able to go about in a short time without inconvenience or fear of injury. Both these advantages are, it appears to me, in great degree, if not wholly, imaginary; at all events, I have never, in a single instance, witnessed the disagreeable gaping spoken of by Dr. Davenport, much less any violent inflammation, or the formation of purulent matter. On the other hand, it is always attended with incomparably greater difficulty of execution, its results are more uncertain, from the impossibility of determining what parts have been divided, and lastly, it is invariably followed by extensive ecchymosis, not only of the eye, but in many cases also of the lids. For these and other reasons that might be assigned, I would discourage the adoption of Mons. Guerin's operation.

The operative procedures above described have more especial reference, it will be perceived, to convergent strabismus; with slight modifications, however, they are equally applicable to the other forms of the lesion. From the more exposed situation of the ball of the eye, the outer straight muscle is much more easily approached and divided than the internal; as to the relative facility of operating on the upper and lower, I can say very little from personal experience, but should suppose the difference, if any, to be trifling. Nor have I had occasion to cut either of the oblique muscles, and from what knowledge I possess on the subject, I am greatly disposed to doubt whether they have much, if any, agency in the production of strabismus. In several instances in which these fasciculi were divided by Lucas, Calder, and others, no impression whatever seems to have been made upon the distortion, and nearly all surgeons agree in the opinion that they should not be interfered with.

Attempts have been recently made, both in this country and in Europe, to disparage the operation for strabismus, on the ground tnat the eye has a tendency to return, in a short time, to its original malposition, or that a new deviation will sometimes occur. No proof, however, of such a result, founded upon an adequate number of statistical facts, has yet been given to the profession. On the contrary, we have reason to believe, from the results that have been furnished by different surgeons, that the reverse usually happens, the operation, instead of being useless, as many have asserted, being generally attended with the most complete success. From most of my own cases being scattered over the country, I have received authentic information, as to the results of the operation, from ten only, of which eight are perfectly successful, a year or more having elapsed since the operation was performed on some of them. In these ten cases both the internal straight muscles were divided in six, in the other four one only. The former have all turned out favorably; of the latter, two have partially failed, or, rather, they have been successful as far as concerns the eye that was most affected, and the only one operated upon. Within the last few months, Dr. J. B. Dixon,* of Norwich, England, has published a list of forty-one cases of convergent strabismus, treated by division of the internal straight muscles, and which one year afterwards presented the following results: in thirty-one, both eyes were perfectly natural; in five, where one organ alone was operated upon, there was slight obliquity of the other; in two cases the squint was changed to a leer; and in three others the eye returned to its original malposition. These results are in the highest degree gratifying, and they are sufficient to show that the operation for strabismus deserves to be ranked among the established resources of surgery, which seldom exhibits such an amount of

* Boston Medical and Surgical Journal, Dec. 29, 1842.

successful terminations. Let me close the discourse, gentlemen, by laying down this maxim for your guidance in regard to the subject we have been considering—"*Never operate on one eye only, when you are certain, as you may be in ninety-nine cases out of a hundred, that both are affected.*"

